# DST-SGR: a Mamba-inspired dual-stream spectro-temporal network for remaining useful life prediction of rotating machinery

**DOI:** 10.3389/frai.2026.1857281

**Published:** 2026-09-22

**Authors:** A. S. Gaurish Kumar, S. Madhavan, R. Dhanalakshmi

**Affiliations:** 1 School of Computer Science and Engineering, Vellore Institute of Technology, Chennai, Tamil Nadu, India; 2 Centre for Cyber Physical Systems, School of Computer Science and Engineering, Vellore Institute of Technology, Chennai, Tamil Nadu, India

**Keywords:** bearing fault diagnosis, remaining useful life (RUL) prediction, Mamba-inspired architecture, state space models (SSMs), gated recurrent networks, predictive maintenance, time-frequency analysis

## Abstract

**Introduction:**

Predicting the Remaining Useful Life (RUL) of rolling bearings is essential in predictive maintenance. High-frequency vibration signals present a dual-domain challenge, since impulses are most visible in the time domain while harmonics are most visible in time-frequency analysis. Existing approaches are limited by vanishing gradients in LSTM networks and by the quadratic O(L^2^) cost of transformer models.

**Methods:**

We introduce DST-SGR, a Mamba-inspired dual-stream spectro-temporal network in which selective temporal modeling is realized through optimized gated recurrent (GRU-based) state interactions with linear O(L) computational cost in a fully cross-platform PyTorch implementation. A temporal branch detects impulses from raw time-series data while a spectral branch learns harmonic signatures from an STFT spectrogram, and three Spectro-Temporal Interaction Blocks (STIBs) couple the streams through bidirectional cross-gated fusion.

**Results:**

On the NASA IMS bearing dataset, under a leakage-free, file-level evaluation protocol with piecewise RUL labels, DST-SGR attains a test-set MAE of 0.152 and RMSE of 0.191, with a five-fold file-level cross-validation MAE of 0.128 ± 0.004, and generalizes to IMS Tests 2 and 3 under changed fault locations.

**Discussion:**

The model contains 131,137 trainable parameters and trains in approximately 0.97 h on an NVIDIA RTX 4060 GPU, showing that competitive prognostic accuracy is achievable with modest computational resources.

## 1 Introduction

Rotation equipment such as wind turbines, turbine aerodynamic engines, compression machines, and high-speed trains forms an important part of contemporary industrial applications. The essential mechanical components in all these applications include the rolling element bearing (REB), which helps to carry the load and ensures that the movements are smooth. Although their structure is relatively simple, they contribute to about 40–50% of failure cases involving electrical motors (Randall and Antoni, 2011). Even a minor defect can quickly lead to catastrophic consequences.

The financial implications of such accidents are quite significant, given that the cost of industrial breakdowns can amount to thousands of dollars per minute and that the global cost for maintenance is in the billions of dollars each year. There is therefore a shift away from maintenance techniques based on reaction and prevention toward Condition-Based Maintenance (CBM) and Predictive Maintenance (PdM) (Jardine et al., 2006). One aspect of PdM involves determining the remaining useful life (RUL), which refers to how long a system will last before it fails. Vibration signals collected from accelerometers provide rich information about bearing health. However reliable RUL estimation using high-frequency vibration data remains challenging due to several factors.

## 2 Challenges

### 2.1 Dual-domain nature of degradation

The wear of bearing occurs both in time and in frequency domain. In time domain, the appearance of spall will produce an impulse signal with characteristic bearing faults frequency (for example, BPFO, BPFI). The impulse signal is generally weak, non-stationary, and mixed with noise. In the frequency domain, the damage of the bearing will result in the variations of harmonic structures, with the generation of bearing fault frequencies and sideband structures. It is necessary for RUL prediction to model in both domains simultaneously.

### 2.2 Long sequence modeling

The vibration monitoring system used in industries operates at high sampling frequencies like 20 KHz. It results in large sequences of the dataset even with small windows of observation. To detect wear and tear properly, the dataset needs to be larger. Traditional modeling techniques face problems in handling such large datasets. The problem is that of vanishing gradient and memory issues in LSTM and GRU whereas Transformers have a computational complexity of O(L2).

### 2.3 Non-stationarity and data scarcity

The deterioration of bearings is by nature a non-stationary process since its statistical features change with time due to changing loading and operating conditions such as loading, speed, and temperature. Also, publicly available bearing data sets like NASA IMS and PRONOSTIA are comparatively small-sized (Qiu et al., 2006; Nectoux et al., 2012).

### 2.4 Limitations of existing methods

The traditional technique using hand-crafted features like Root Mean Square, kurtosis, and other spectral features requires substantial domain knowledge and flexibility. The CNN model is efficient in identifying local feature learning, but it cannot capture the long-range dependencies required by the problem of RUL estimation. Depth is increased to increase the receptive field, which leads to high computational costs and instability in training.

Recurrent models like LSTM and GRU offer sequential modeling but face challenges in memory constraints and vanishing gradients while handling long sequences. State Space Models (SSMs), especially Mamba, have recently developed efficient models using linear computational complexity. While they address scalability issues, they operate solely in the time domain and fail to exploit important spectral information critical for bearing fault analysis.

### 2.5 Proposed approach

To overcome these shortcomings, we introduce the **Dual-Stream Spectro-Temporal Network (DST-SGR)**, an end-to-end model that can be used for predicting RUL. DST-SGR uses two separate processing streams, namely,

- **Temporal Stream:** extracts information from the raw waveform signal of the vibrations (e. g., 2,048 points).
- **Spectral Stream:** generates spectrograms using Short- Time Fourier Transform with a window of 64 and hop length of 16.

They both are modeled via Selective Gated Recurrent (SGR) blocks (Mamba-inspired) whose computational complexity is linear in O(L). To facilitate interaction between them, Spectro- Temporal Interaction Blocks (STIBs) are proposed, in which bidirectional cross gated fusion helps transfer features from one modality to another. Temporal features are adaptively sampled for aligning with spectral features, whereas spectral features are up sampled for correlating impulsive events with their spectral signature.

Finally, features from both streams are globally pooled, concatenated, and passed through a Multi-Layer Perceptron (MLP) regression head trained using Huber loss for RUL estimation.

### 2.6 Contributions

The key contributions of our work include:

- **Dual-stream architecture:** we introduce DST-SGR, a new dual-stream architecture, that leverages the combination of time-domain features and time-frequency domain representations using Mamba-inspired, linear-complexity Selective Gated Recurrent blocks for efficient representation of long sequences.
- **Cross-gated fusion mechanism:** we propose Spectro- Temporal Interaction Block (STIB), which facilitates bi- directional communication between streams.
- **Comprehensive evaluation:** the proposed approach evaluated under a leakage-free file-level protocol with five-fold cross-validation on IMS Test 1, cross-condition evaluation on IMS Tests 2 and 3, and comparison against CNN, LSTM, GRU, and Transformer baselines trained under an identical protocol.

## 3 Literature survey

Prediction of Remaining Useful Life (RUL) is a fundamental task in Condition-Based Maintenance (CBM) and Predictive Maintenance (PdM). Over the past decades, methodologies have evolved from physics-based models and signal processing techniques to modern deep learning approaches capable of learning hierarchical degradation features directly from raw data (Zhao et al., 2019).

### 3.1 Conventional signal processing and physics-based prognostics

In earlier times, the methods that were utilized for predicting RUL included the use of hand-crafted features from vibration data. RMS (root mean square), kurtosis, crest factor, and skewness were some of the common parameters used. Kurtosis is an interesting parameter since it is very sensitive to impulse fault diagnosis (Antoni, 2006).

Harmonic patterns corresponding to specific failures were found using FFT-based frequency domain techniques, hence providing more detailed diagnostic information. Envelope analysis helped diagnose problems earlier because it could extract repetitive impulses from wideband signals. The advanced techniques used were spectral kurtosis and kurtogram techniques that allowed choosing appropriate frequency bands (Antoni and Randall, 2006; Randall and Antoni, 2011).

Wavelet transform approaches, which consist of Continuous Wavelet Transform (CWT) and Wavelet Packet Decomposition (WPD), enabled time-frequency analysis, thus enabling the simultaneous localization of transients. Nonetheless, wavelet transform approaches rely largely on the choice of parameters, such as the mother wavelet and decomposition level (Peng and Chu, 2004; Feng et al., 2013).

Models based on physics tried to incorporate degradation phenomena by modeling crack propagation along with stochastic filtering techniques like Kalman Filter and Particle Filter (Saxena et al., 2008; Lei et al., 2018). Although conceptually sound, such approaches necessitate extensive information about the system and cannot be generalized easily.

### 3.2 Convolutional neural networks for bearing prognostics

The CNN model made it possible to extract features automatically from raw vibration data without the requirement for manual feature selection. It was found that 1D-CNN could learn features related to the fault like conventional techniques (Sateesh Babu et al., 2016; Li et al., 2018).

It was possible to improve the performances of these CNN models owing to their ability to recognize both local and global patterns. However, there is no sequence memory in the CNN models, and they are limited by their receptive field that makes it difficult for them to model the long-term trend in estimating RUL.

### 3.3 Recurrent architectures and sequence modeling

RNNs, especially LSTM networks, were widely used for RUL prediction owing to their capability to capture temporal dependencies (Hochreiter and Schmidhuber, 1997; Heimes, 2008). LSTM networks can effectively learn the degradation evolution process and are superior to convolutional neural network models in handling sequential data (Zheng et al., 2017; Guo et al., 2017).

Further improvements were made using techniques such as Bi-LSTM and hybrid CNN-LSTM, where spatial and temporal aspects were combined (Zhang et al., 2018).

However, recurrent models have some weaknesses:

- Vanishing gradient problem when handling long sequences.
- Sequential processing that restricts parallelization.
- Fixed hidden state size leading to memory issues.

### 3.4 Transformer-based prognostic models

The transformer network structures have proposed attention-based architecture that facilitates the modeling of long dependencies without recurrence. These networks have shown good results in sequence modeling problems such as Remaining Useful Life estimation (Vaswani et al., 2017; Mo et al., 2021).

But one major limitation is the complexity associated with them. The complexity of these algorithms is of the order O(L2) for time and space. This makes them unsuitable for vibration signals having high frequency and large sequence lengths

### 3.5 State space models and Mamba

The State Space Model (SSM) is one such model, where S4 and its various extensions have shown great promise as models that scale better than Transformer models in processing sequences (Gu et al., 2022). The Mamba framework is an improvement over SSMs with state transition selections and linear complexity O(L) (Gu and Dao, 2023). Mamba has shown its proficiency in sequence modeling and has been rapidly adopted across domains ranging from medical image analysis to image restoration (Xiao et al., 2025; Liu et al., 2025; Wang et al., 2025; Xiong et al., 2025; Zhu et al., 2025). Nonetheless, the traditional Mamba method is exclusively confined to time domain features without any provision to account for frequency domain features such as harmonics, side bands, and spectral changes required for bearing condition assessment.

## 4 Research gap

However, despite considerable progress in predicting the RUL of bearings, various shortcomings still exist. These shortcomings include the inability of the existing methods to consider the following at the same time:

- Combining time domain and frequency domain degradation signatures.
- A linear computation process of the vibration signal at high frequencies over a long period of time.
- Inter play between the two domains correlating time domain impulses to their frequency domain counterparts.

The CNN models cannot handle global sequence modeling, while the LSTM models have problems with vanishing gradient and sequential computing, and the Transformer models require an unacceptably large amount of computation that grows quadratically with the length of the sequences. While Mamba allows linear scalability, it works only in the time domain.

This gap motivates the need for a unified architecture that can efficiently capture both temporal and spectral characteristics while maintaining scalability for long sequence industrial data.

## 5 Materials and methods

The Dual Stream Spectro Temporal network (DST-SGR) framework proposed here attempts to take into consideration the dual domain property associated with bearing deterioration to make effective RUL predictions. Defects in bearings appear to occur impulsively in the time domain while there are harmonic changes occurring in the frequency domain.

To address the above limitations, the DST-SGR framework includes the following three components:

- Dual-stream processing to represent temporal and spectral information separately.
- Mamba-inspired Selective Gated Recurrent (SGR) blocks to achieve efficient long sequence modeling.
- Bidirectional cross-domain interaction via Spectro-Temporal Interaction Blocks (STIBs).

The overall architecture consists of three modules: (i) Input Stem for dual-domain embedding, (ii) Dual-Stream Backbone composed of stacked STIBs, and (iii) Prediction Head for RUL regression.

### 5.1 Overall architecture overview

The novel Dual-Stream Spectro-Temporal Network (DST-SGR) architecture is an end-to-end deep learning framework specially developed to predict the Remaining Useful Life (RUL) of rolling element bearings in operational conditions in industry. The main motivation behind such design comes from the duality of bearing degradation where surface defects and fatigue cracks lead to transient impulse events in the time domain signal, whereas progressive damage leads to systematic changes in the spectral energy distribution, particularly by increasing the amplitudes of harmonics (e.g., BPFO, BPFI, and BSF) along with the appearance of rotational sidebands. To address this problem, DST-SGR utilizes dual-stream architecture, which uses two separate processing streams. The temporal stream captures the impulse-based pattern from the time domain.

To enable effective learning of cross-domain relationships, the architecture incorporates multiple Spectro-Temporal Interaction Blocks (STIBs), which facilitate bidirectional information exchange between temporal and spectral streams at each layer. This interaction allows the model to correlate impulsive events with their corresponding spectral signatures throughout the degradation process.

The final RUL prediction is obtained by aggregating features from both streams using global pooling, followed by feature fusion and regression through a Multi-Layer Perceptron (MLP).

The overall DST-SGR architecture, illustrated in Figure 1, consists of five stages:

- **Step 1 Input stem:** temporal and spectral embedding of input vibration signals.
- **Step 2 Dual stream back-bone:** *N* = 3 Spectro-Temporal Interaction Blocks (STIBs) with parallel SGR-based sequence modeling and cross-gating of feature interaction.
- **Step 3 Feature aggregation:** Independent Global Average Pooling applied to both the temporal and spectro-temporal streams.
- **Step 4 Feature fusion:** aggregated feature vectors concatenated into a single representation.
- **Step 5 Regression head:** multi-layer Perceptron (MLP)-based decoding for estimating the RUL.

**Figure 1:**
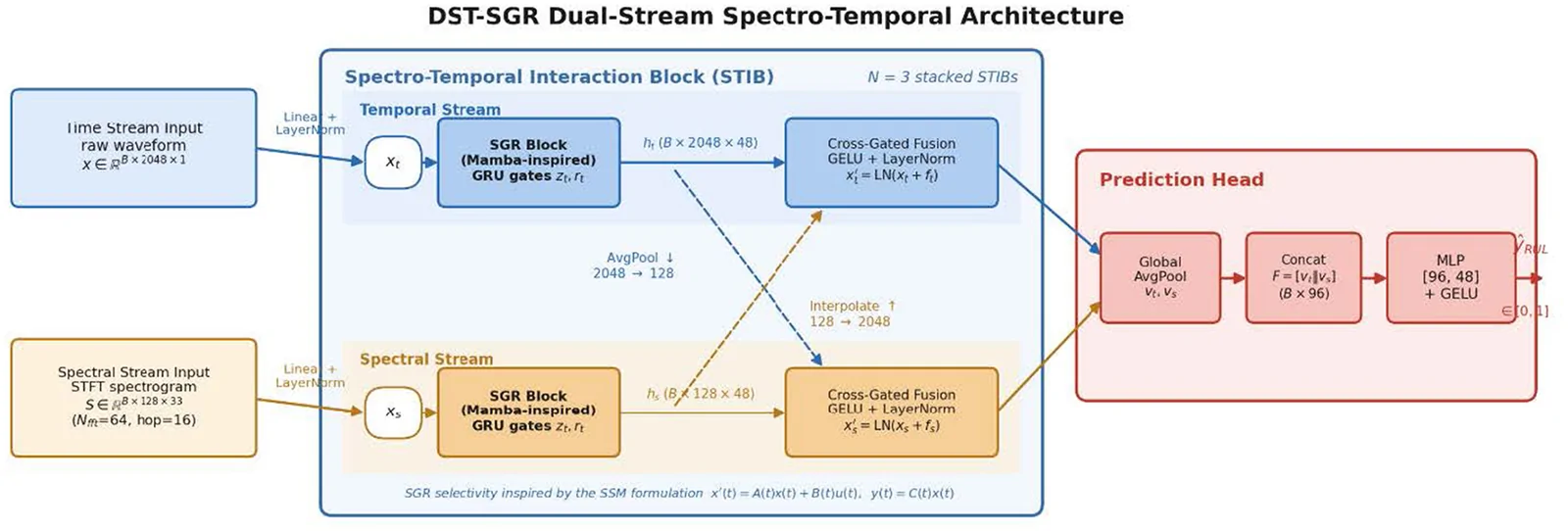
DST-SGR dual-stream spectro-temporal architecture.

The complete architecture and hyperparameter configuration is summarized in Table 1.

**Table 1:** DST-SGR architecture and hyperparameters.

Parameter	Value
Input size	2,048
Sampling rate	20kHz
Model dimension	48
STIB layers	3
SSM state dimension	12
FFT size	64
Hop length	16
Spectrogram size	128 × 33
MLP dims	[96, 48]
Dropout	0.1
Parameters	131,137

### 5.2 Dataset description

The DST-SGR model is evaluated using the NASA IMS Bearing dataset, one of the most widely used benchmark datasets for bearing degradation analysis under realistic operating conditions. The data set was obtained by the University of Cincinnati Center for Intelligent Maintenance Systems (IMS) under an experimental setting that mimicked an industrial environment of bearings (Qiu et al., 2006). The experimental arrangement involves four Rexnord ZA-2115 double row bearings installed on a rotating shaft operating at a constant speed of 2,000 RPM with a radial load of 6,000 lbs imposed by means of a spring load.

Vibration data is acquired at a sampling frequency of 20,480 Hz (approximately 20 kHz). Following common practice for this dataset, the nominal 20 kHz value is used in spectral-resolution calculations. Each recording contains 1 s of vibration data, corresponding to 20,480 samples per channel, collected at 5-min intervals for the first 43 records and at 10-min intervals thereafter.

The dataset includes three experimental runs, referred to as Test1, Test2, and Test3. In this study, Test 1 is utilized, which spans approximately 34 days of operation (22 October to 25 November 2003). During this period, Bearing3 develops an outer race fault, whileBearing4 exhibits rolling element defects, providing a complete run-to-failure degradation trajectory suitable for RUL prediction.

For model training, the raw vibration signals are segmented into non-overlapping windows of length *L* = 2,048 samples, and each window is standardized (zero mean, unit variance) per recording. RUL labels follow a piecewise degradation model: the label remains 1.0 throughout the healthy stage and decreases linearly to 0.0 between the First Prediction Time (FPT) and failure, reflecting the non-linear plateau-then-degrade behavior of real bearings (Section 6.2).

The input samples used for training the DST-SGR model consist of single-channel windowed segments of vibration signals obtained from accelerometer readings. The input tensor is defined in Equation 1:


$$\begin{array}{l} X\in R^{\left\{B\times 2048\times 1\right\}} \end{array}$$ (1)

where *B* denotes the batch size, 2,048 represents the window length, and 1 corresponds to the single vibration amplitude channel.

The window length of *L* = 2,048 samples corresponds to approximately 0.1 s of vibration data sampled at 20 kHz. This choice balances two competing requirements. First, it ensures that multiple fault-induced impulse events are captured within each window. For instance, at a Ball Pass Frequency Outer (BPFO) of approximately 236 Hz (for IMS bearings operating at 2,000 RPM), one impulse occurs roughly every 87 samples, allowing for the inclusion of several impulses in one window. Secondly, it keeps the length of the sequence within reasonable limits to ensure efficiency during training in the SSM-based framework.

While traditional spectrogram-based models rely on the complete transformation of the signal before feeding it into the model, DST-SGR operates directly on raw time domain signals of a single channel. The corresponding spectral representations are learned automatically inside the STFT module embedded in the network architecture.

### 5.3 Spectro-temporal feature stem

In the Input Stem, the input signal X is split into two streams, each having an individual embedding process for obtaining two streams with different representations of degradation. These two streams are mapped into the same embedding space with *D* = 48 dimensions.

#### 5.3.1 Temporal stream embedding

The temporal stream embeds the raw waveform into a high-dimensional space using a linear projection followed by normalization, as defined in Equation 2. The embedding is done in the native resolution of the signal, meaning that no temporal down sampling is done to retain the full number of 2,048 timesteps. This decision is important since impulsive events in case of bearing faults can last for less than 10 timesteps.


$$\begin{array}{l} x\text{\_}t=LayerNorm\left(W\text{\_}t\;x\;+\;b\text{\_}t\right),\;x\text{\_}t\;\in \;{\mathbb{R}}^{B\;\times \;2048\;\times \;D} \end{array}$$ (2)

Normalization stabilizes the distribution of features and improves convergence during early training epochs when embedding parameters are randomly initialized. The resulting temporal representation is given in Equation 3:


$$\begin{array}{l} x_{t}\;\in \;R^{\left\{B\;\times \;2048\;\times \;48\right\}} \end{array}$$ (3)

This representation serves as input to the temporal SGR branch in the first STIB module.

#### 5.3.2 Spectral stream embedding

The spectral stream uses a trainable Short-Time Fourier Transform (STFT) to obtain the spectrogram of the wave, as described in Equations 4–8. The STFT parameters are:


$$\begin{array}{l} N_{FFT}=64,\;H=16,\;W=64 \end{array}$$ (4)

These settings yield a frequency resolution:


$$\begin{array}{l} \text{Δ }f=\frac{20000}{64}=\;312.5\;Hz/bin \end{array}$$ (5)

and temporal frame rate:


$$\begin{array}{l} \frac{20000}{16}=\;1250\;frames/s \end{array}$$ (6)

The resulting spectrogram is:


$$\begin{array}{l} S\;\in \;R^{\left\{B\;\times \;128\;\times \;33\right\}} \end{array}$$ (7)

This is adequate in terms of resolution for identifying fault harmonics such as BPFO (236 Hz) and BPFI (297 Hz), as well as having adequate time resolution for recording the modulating pattern in amplitudes caused by dynamics in the loading zone.

The spectrogram is then mapped into the embedding space using a linear encoder with layer normalization (Ba et al., 2016), yielding the spectral representation in Equation 9:


$$\begin{array}{l} x\text{\_}s=LayerNorm\left(W\text{\_}s\;X\text{\_}s\;+\;b\text{\_}s\right),\;x\text{\_}s\;\in \;{\mathbb{R}}^{B\;\times \;128\;\times \;D} \end{array}$$ (8)

$$\begin{array}{l} x_{s}\;\in \;R^{\left\{B\;\times \;128\;\times \;48\right\}} \end{array}$$ (9)

The asymmetry of sequence length between temporal and spectral streams (2,048 vs. 128) stems from the tradeoff dictated by the Heisenberg uncertainty principle during time frequency analysis. The temporal stream exhibits high time resolution but lacks spectral information, while the spectral stream exhibits improved frequency resolution but poor temporal resolution.

Despite this difference, both streams share the same embedding dimension *D* = 48, ensuring compatibility for cross-stream interaction within the STIB framework.

### 5.4 Dual-stream interaction backbone

The backbone of DST-SGR consists of a stack of *N* = 3 Spectro-Temporal Interaction Blocks (STIBs). Each STIB performs two key operations: (i) independent parallel sequence modeling within each stream using Mamba-inspired Selective Gated Recurrent (SGR) blocks, and (ii) bidirectional cross-stream feature exchange via a cross-gated fusion mechanism. The feature representations are progressively refined across the stacked STIB layers.

#### 5.4.1 Parallel Selective Gated Recurrent (SGR) processing (Mamba-inspired)

Within each STIB, the temporal and spectral streams are processed independently by employing two Selective Gated Recurrent (SGR) blocks (Mamba-inspired) of identical structure but different parameter sets, as defined in Equations 10, 11:


$$\begin{array}{l} h_{t}\;=SGR_{t}\left(x_{t}\right),\;h_{t}\;\in \;R^{\left\{B\;\times \;2048\;\times \;D\right\}} \end{array}$$ (10)

$$\begin{array}{l} h_{s}=SGR_{s}\left(x_{s}\right),\;h_{s}\;\in \;R^{\left\{B\;\times \;128\;\times \;D\right\}} \end{array}$$ (11)

The design is inspired by the selective state space model formulation:


$$\begin{array}{l} {\text{x}}^{\prime }\left(t\right)=A\left(t\right)x\left(t\right)+B\left(t\right)u\left(t\right),y\left(t\right)=C\left(t\right)x\left(t\right) \end{array}$$

with time-dependent matrixes *A(t), B(t)*, and *C(t)*. In DST-SGR this selectivity is implemented by a gated recurrent approximation rather than the official Mamba parallel-scan kernel. Each SGR block applies layer normalization, a depth wise 1-D convolution for local context, and a GRU (Cho et al., 2014; Chung et al., 2014) whose update and reset gates, defined in Equations 12–15,


$$\begin{array}{l} z\text{\_}t=\sigma \left(W\text{\_}z\;x\text{\_}t\;+\;U\text{\_}z\;h\text{\_}\left\{t-1\right\}\right), \end{array}$$ (12)

$$\begin{array}{l} r\text{\_}t=\sigma \left(W\text{\_}r\;x\text{\_}t\;+\;U\text{\_}r\;h\text{\_}\left\{t-1\right\}\right), \end{array}$$ (13)

$$\begin{array}{l} \tilde{h}\text{\_}t=tanh\left(W\text{\_}h\;x\text{\_}t\;+\;U\text{\_}h\;\left(r\text{\_}t\;⊙\;h\text{\_}\left\{t-1\right\}\right)\right) \end{array}$$ (14)

$$\begin{array}{l} h\text{\_}t=\left(1-z\text{\_}t\right)\;⊙\;h\text{\_}\left\{t-1\right\}\;+\;z\text{\_}t\;⊙\;h˜\text{\_}t \end{array}$$ (15)

provide input-dependent control of information flow, followed by a residual connection. We emphasize that GRU and Mamba differ in mathematical formulation, training parallelization, and gradient propagation; the SGR block reproduces the input-dependent selectivity and linear complexity of Mamba, but not its parallel-scan computation. A comparison against the official Mamba CUDA kernels is left as future work.

In the SGR block, the dimension of the state is d_state = 12, the kernel size of the convolution is d_conv = 4, and the expansion rate is 2; thus, the intermediate dimension is determined as D_inner = 96, followed by projecting it back to *D* = 48. Sequential gated recurrence over cuDNN-optimized kernels yields linear O(L) complexity for both streams (without the parallel-scan training speedup of official Mamba kernels).

#### 5.4.2 Bidirectional cross-gated fusion

Following parallel SGR processing, cross-gated fusion enables explicit interaction between temporal and spectral representations.

#### 5.4.3 Temporal to spectral fusion

Temporal features undergo adaptive average pooling for down sampling, and the fused spectral representation is obtained as given in Equations 16–18:


$$\begin{array}{l} {\tilde{h}}_{t}\;\in \;R^{\left\{B\;\times \;128\;\times \;D\right\}} \end{array}$$ (16)

Spectral feature fusion is obtained from:


$$\begin{array}{l} f_{s}=GELU\left(W_{fs}\left[x_{s}\;||{\;\tilde{h}}_{t}\right]\;+\;b_{fs}\right) \end{array}$$ (17)

$$\begin{array}{l} x_{s}^{\prime }=\;LayerNorm\left(x_{s}+f_{s}\right) \end{array}$$ (18)

This maintains the average energy distribution of temporal features, thereby making the correlation between impulse energy and spectral energy possible.

#### 5.4.4 Spectral-to-temporal fusion

Similarly, spectral features are upsampled to match temporal resolution, and the temporal stream is updated as given in Equations 19–21:


$$\begin{array}{l} {\tilde{h}}_{s}\;\in \;R^{\left\{B\;\times \;2048\;\times \;48\right\}} \end{array}$$ (19)

The bidirectional interaction allows the model to associate temporal impulse events with corresponding spectral charac- teristics and vice versa. Dropout with probability *p* = 0.1 is applied within fusion layers to improve generalization.


$$\begin{array}{l} f_{t}=GELU\left(W_{ft}\left[x_{t}\;||{\;\tilde{h}}_{s}\right]\;+\;b_{ft}\right) \end{array}$$ (20)

$$\begin{array}{l} x_{t}^{\prime }=\;LayerNorm\left(x_{t}+f_{t}\right) \end{array}$$ (21)

### 5.5 Feature pooling layer

The outputs from the final STIB model for the temporal and spectral streams, respectively denoted by $x_{t}\;\in \;R^{\left\{B\;\times \;2048\;\times \;48\right\}}\;$and $x_{s}\;\in \;R^{\left\{B\;\times \;128\;\times \;48\right\}}$, are projected to have fixed size representations, as defined in Equations 22, 23.

#### 5.5.1 Temporal pooling


$$\begin{array}{l} p_{t}=AvgPool\left(x_{t}\right),p_{t}\;\in \;R^{\left\{B\;\times \;48\right\}} \end{array}$$ (22)

#### 5.5.2 Spectral pooling


$$\begin{array}{l} p_{s}=AvgPool\left(x_{s}\right),p_{s}\;\in \;R^{\left\{B\;\times \;48\right\}} \end{array}$$ (23)

The global average pooling operation computes the mean activation across all time steps in each stream, resulting in a compact 48-dimensional representation that captures the average degradation state over the observation window.

This approach is preferred over global max pooling, which captures only peak activations while ignoring overall signal trends, and over flattening, which leads to excessively high- dimensional representations (2,048 × 48 + 128 × 48 = 104, 448 dimensions) unsuitable for efficient regression.

The pooled features from both streams are concatenated to form the final feature vector given in Equations 24, 25:


$$\begin{array}{l} F=\left[p_{t}\;||\;p_{s}\right]\;\in \;R^{\left\{B\;\times \;96\right\}} \end{array}$$ (24)

This fused representation captures both the time-averaged impulse energy profile from the temporal stream and the time-averaged harmonic evolution from the spectral stream, providing a comprehensive description of the degradation state for RUL prediction.

### 5.6 Regression prediction head

The fused feature vector


$$\begin{array}{l} F\;\in \;R^{\left\{B\;\times \;96\right\}} \end{array}$$ (25)

is passed to a two-layer Multi-Layer Perceptron (MLP) regression head to produce the normalized Remaining Useful Life (RUL) prediction.

The MLP consists of two fully connected layers with hidden dimensions [96, 48] and employs Gaussian Error Linear Unit (GELU) activation after each hidden layer, as defined in Equations 26–28:


$$\begin{array}{l} h_{1}=GELU\left(W_{1}F\;+\;b_{1}\right) \end{array}$$ (26)

$$\begin{array}{l} h_{2}=GELU\left(W_{2}h_{1}\;+\;b_{2}\right) \end{array}$$ (27)

$$\begin{array}{l} ŷ=W_{3}\;h_{2}\;+\;b_{3} \end{array}$$ (28)

GELU activation (Hendrycks and Gimpel, 2016) is chosen over ReLU due to its smooth non-linear behavior and non-zero gradients for negative inputs, which helps avoid neuron inactivity during early training. Drop out with probability *p* = 0.1 is applied after each hidden layer to improve generalization and reduce overfitting.

The final output $\tilde{y\;}$is a scalar representing normalized RUL in the range [0, 1], where 1 corresponds to a fully healthy bearing and 0 indicates imminent failure. No activation function is applied at the output layer, allowing the model to learn an unconstrained regression mapping. Accordingly, no clipping to [0, 1] is applied at inference, and all reported predictions and metrics use the raw outputs (see Section 6.3).

### 5.7 Training strategy and optimization

The DST-SGR model is trained end-to-end using the Huber loss function (Huber, 1964) with δ = 1.0, defined in Equations 29, 30:


$$\begin{array}{l} L_{\delta }=\frac{1}{2}{\left(\;ŷ\;-\;y\right)}^{2},\;|\;ŷ\;-\;y\;|\;\leq \;\delta \end{array}$$ (29)

$$\begin{array}{l} \delta \;\left(|\;ŷ\;-\;y|\;-\;\frac{1}{2}\;\delta \right),\;otherwise \end{array}$$ (30)

Huber loss combines the advantages of both L1 and L2 loss, in which it behaves like MSE with small residuals and MAE with large residuals. This makes it more suitable for predicting RUL from run-to-failure datasets such as NASA IMS, which contains non-monotonic trends and outliers, especially at the initial and final states of good and bad health, respectively. Network training is carried out using the AdamW optimizer (Loshchilov and Hutter, 2019) with the fused version for better efficiency. Hyperparameters used during training are listed below:

- Learning rate: η = 1 × 10^−4^
- Weight decay: λ = 1 × 10^−5^
- Exponential decay rates: β1 = 0.9, β2 = 0.999

The fused AdamW implementation leverages CUDA kernel fusion to execute parameter updates in a single GPU operation, resulting in approximately 15–20% faster training compared to standard implementations. The decoupled weight decay mechanism ensures consistent regularization across model parameters independent of gradient scaling.

A Reduce LROn Plateau scheduler is employed to adaptively adjust the learning rate based on validation performance. The learning rate is reduced by a factor of 0.5 if no improvement in validation loss is observed for 5 consecutive epochs. This enables rapid convergence during early training stages followed by fine-tuning as the model approaches optimal performance. Early stopping with a patience of 15 epochs is used to prevent overfitting by terminating training when validation loss Ceases to improve.

The training procedure is performed for up to 100 epochs using a batch size of *B* = 48, ensuring a trade-off between efficient use of memory and consistent gradient computation based on the limitation of 8 GB of VRAM on the NVIDIA RTX 4060.

Moreover, the following hardware-level optimization flags are set to enhance training performance:

- torch.backends.cudnn.benchmark = True.
- torch.backends.cuda.matmul.allow_tf32 = True.

### 5.8 Computational complexity analysis

The computational efficiency of DST-SGR is primarily derived from the linear complexity of the Mamba-inspired Selective Gated Recurrent architecture.

The temporal stream operates with complexity:


$$\begin{array}{l} \text{O}\left(\text{L}\right)=\text{O}\left(2048\right) \end{array}$$

The spectral stream operates with:


$$\text{O}(\text{T})\text{=O}(\text{128})\text{=O}(\frac{\text{L}}{\text{16}})$$

Cross-gated fusion operations within each STIB involve pooling, interpolation, and linear projections:


$$\begin{array}{l} \text{O}\left(\text{T}\right)+\text{O}\left(\text{L}\right)=\text{O}\left(\text{L}\right) \end{array}$$

Global average pooling in the feature aggregation stage also incurs:


$$\begin{array}{l} \text{O}\left(\text{T}\right)+\text{O}\left(\text{L}\right)=\text{O}\left(\text{L}\right) \end{array}$$

The MLP regression head operates on fixed-size vectors (2D = 96), resulting in constant complexity:


$$\begin{array}{l} \text{O}\left(1\right) \end{array}$$

Thus, the overall computational complexity of DST-SGR is:


$$\begin{array}{l} \text{O}\left(\text{N}\cdot \text{L}\right) \end{array}$$

Where N=3 denotes the number of STIB layers. This is significantly more efficient than Transformer-based architecture, which requires:


$$\begin{array}{l} \text{O}\left(\text{N}\cdot \text{L}2\right) \end{array}$$

For self-attention operations. For a sequence length of *L*= 2,048, this results in a substantial computational reduction.

The full training pipeline including STFT preprocessing, dual-stream forward passes, Huber loss computation, and AdamW parameter updates completes at approximately 0.111 s per batch (batch size 48), corresponding to 9.0 iterations per second and 432 samples per second on an NVIDIA RTX 4060 Laptop GPU (8 GB VRAM). Total training time to convergence across 100 epochs was approximately ~0.97 h (~58 min), confirming the practical deployability of DST-SGR on consumer-grade hardware without requiring cloud infrastructure.

## 6 Results and discussion

### 6.1 Experimental setup

Our proposed dual-stream architecture is further assessed using the NASA IMS (Intelligent Maintenance Systems) bearing dataset, particularly the first test subset, which captures the complete run-to-failure lifecycle of four Rexnord ZA-2115 double-row rolling element bearings mounted on a single shaft. During the experiment, the system operated continuously at a constant speed of 2,000 RPM under a radial load of 6,000 lbs.

Vibration signals were acquired using piezoelectric accelerometers mounted radially on each bearing housing and recorded via a National Instruments data acquisition at a sampling frequency of 20,480 Hz (nominal 20 kHz). The dataset contains 2,156 acquisition instances, with each instance recording 20,480 samples per channel across eight channels (two accelerometers per bearing). The 2,156 acquisitions span the approximately 34-day campaign (22 October to 25 November 2003). Bearing 3, which developed the outer race fault, is monitored by channels 5–6; this study uses its *x*-axis channel.

### 6.2 Data preprocessing

Raw signals for vibration are extracted using temporal windows of length:


$$\begin{array}{l} \text{L}=2,048\text{ samples} \end{array}$$

where each window corresponds to around 102. Four milliseconds of signal at a sampling rate of 20 kHz. At a shaft speed of 2,000 RPM, the number of revolutions captured per window would be 3–4 cycles, thus ensuring that frequencies such as BPFO, BPFI, and BSF are completed at least once in every segment.

Windows are extracted without overlap (stride equal to the window length), so no two windows share any samples.

RUL labels are assigned per acquisition file using a piecewise model. The First Prediction Time (Sutrisno et al., 2012; Li et al., 2015) is detected automatically from per-file health indicators (kurtosis and RMS of the raw signal, median-smoothed) using a 3σ rule over the healthy baseline with a consecutive-exceedance requirement; for Test 1 the FPT occurs at file 634 of 2,156 (29.4% of life). Labels are 1.0 before the FPT and decay linearly to 0.0 at failure (Figure 2: health indicators with detected FPT).

**Figure 2:**
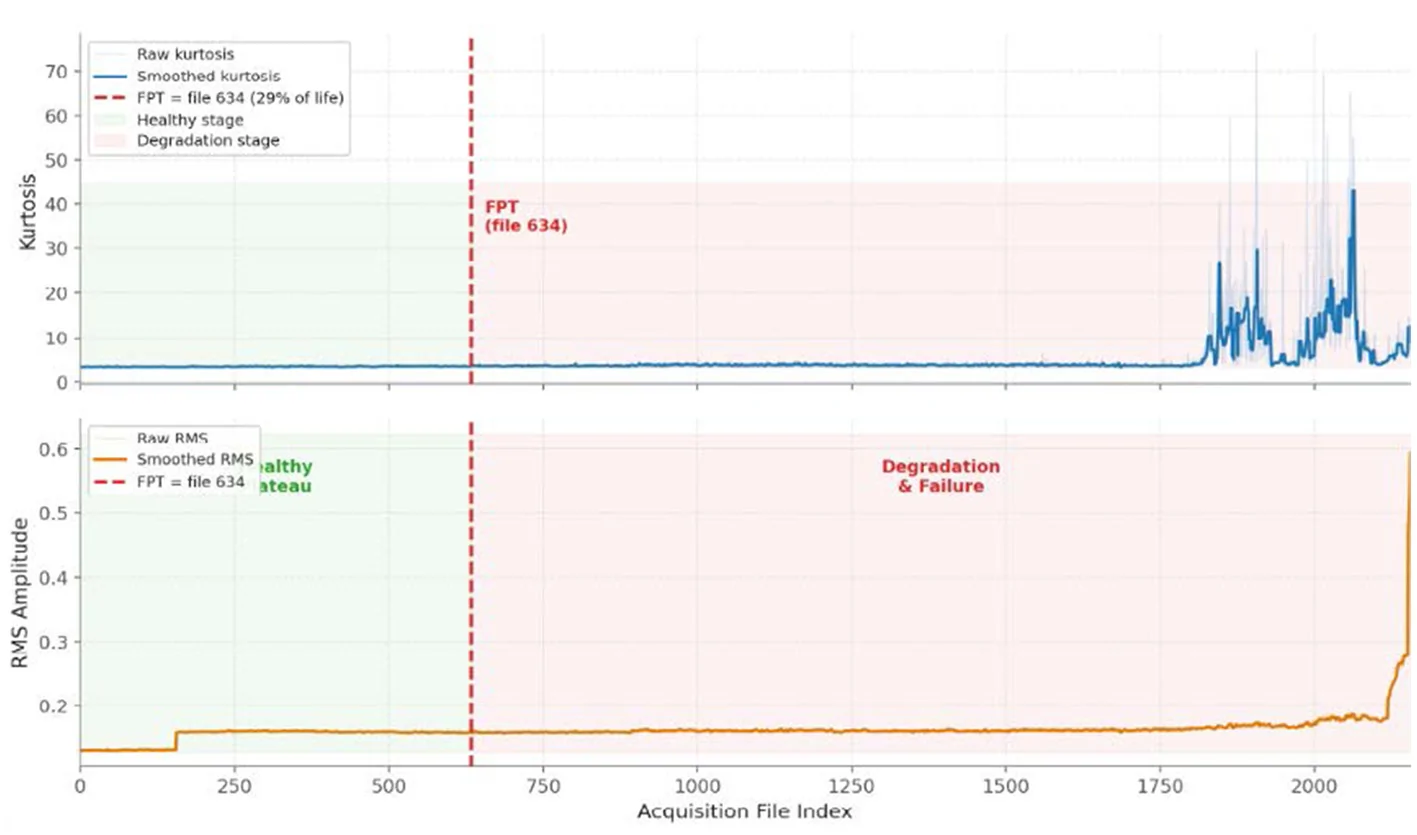
Bearing health indicators for NASA IMS Test 1 (Bearing 3, *x*-axis channel) plotted against acquisition file index. **(Top)** Kurtosis (raw and median-smoothed, window *k* = 5). **(Bottom)** RMS amplitude (raw and median-smoothed). The vertical dashed red line marks the First Prediction Time (FPT) detected at file index 634 of 2,156 (29.4% of the bearing's operational life), defined as the point at which the smoothed kurtosis first exceeds a threshold of 3.0 standard deviations above the healthy-phase mean. Files prior to the FPT (green region) correspond to the healthy plateau; files after the FPT (red region) correspond to active degradation and eventual failure.

All 2,156 acquisition files are used without any health-state-dependent subsampling, leaving the marginal data distribution unaltered. To prevent information leakage, the training/validation/test partition (70/15/15) is performed at the level of whole acquisition files with a fixed random seed: every window extracted from a given recording is assigned to exactly one partition, so no test window shares signal content with training data. The residual class imbalance between the long healthy stage and the short failure stage is handled exclusively inside the training loader by a weighted random sampler (inverse RUL-bin frequency); validation and test sets retain the natural distribution encountered in deployment.

The processed dataset, summarized in Table 2, consists of:

- Training samples: 15,090.
- Validation samples: 3,230.
- Test samples: 3,240.

**Table 2:** Processed dataset statistics—NASA IMS Test 1.

Partition	Samples	Window length	Stride	RUL range
Training	15,090	2,048 (102.4 ms)	2,048	[0.0, 1.0]
Validation	3,230	2,048 (102.4 ms)	2,048	[0.0, 1.0]
Test	3,240	2,048 (102.4 ms)	2,048	[0.0, 1.0]
Total	21,560	–	–	–

### 6.3 Implementation details

The model is implemented in Python 3.13 using PyTorch 2.7.1 with CUDA 11.8 on Windows 11. The selective temporal modeling is realized by the Selective Gated Recurrent (SGR) block described in Section 5.4, a Mamba-inspired, cross-platform PyTorch implementation built on cuDNN-optimized gated recurrence.

The instantiated model contains a total of 131,137 trainable parameters. The architecture consists of:

- **Temporal stream:** Selective Gated Recurrent (SGR) blocks (hidden dimension = 48, state size = 12).
- **Spectral stream:** STFT preprocessing (Nfft = 64, hop = 16) followed by Selective Gated Recurrent (SGR) blocks.
- **Interaction blocks:** *N* = 3 Spectro-Temporal Interaction Blocks with bidirectional cross-gated fusion.
- **Prediction head:** MLP with hidden dimensions [96, 48] Training is performed on an NVIDIA GeForce RTX 4060 Laptop GPU (8GB VRAM) using the following hyper parameters.

The complete set of training hyperparameters is listed in Table 3.

**Table 3:** Training hyper parameters.

Hyperparameter	Value
Optimizer	AdamW(fused CUDA operations)
LearningRate	1 × 10–4
BatchSize	48
Loss function	Huber (δ = 1. 0)
Max epochs	100
Early stopping	15 epochs
LR scheduler	Reduce LR On Plateau (factor = 0.5, patience = 5)

### 6.4 Training process and convergence

a. A naive Python-loop state-space implementation proved impractically slow (≈800 s per batch on a Tesla T4); the SGR formulation on cuDNN kernels reduces this to approximately 0.11 s per batch on the RTX 4060.

#### 6.4.1 Training convergence

The model converged after 71 epochs using early stopping, requiring approximately 0.97 h of total training time. The training and validation loss curves showed smooth convergence without significant overfitting, demonstrating the effectiveness of the regularization strategy.

### 6.5 Quantitative results

To assess the predictive accuracy of the trained DST-SGR model with the highest level of rigor, the model checkpoint at epoch **71** (best validation loss: 0.01858) was tested against an isolated test set of 3,240 windows which were never seen during any phase of training or hyperparameter optimization; the resulting performance is reported in Table 4. The main evaluation criteria used were the Mean Absolute Error (MAE) and the Root Mean Squared Error (RMSE), calculated using the normalized RUL value range [0, 1].

**Table 4:** Quantitative performance—NASA IMS 1st test.

Metric	Value
Mean Absolute Error (MAE)	0.1522
Root Mean Square Error (RMSE)	0.1909
Best validation loss (huber)	0.01858
Total trainable parameters	131,137
Total training time	~0.97 h
Best epoch	71/100

#### 6.5.1 Predicted RUL trajectory analysis

The single most effective way of measuring the operational efficiency of the predictive model is how accurately it reflects the real-life degradation trend throughout the complete lifetime of the bearing. As can be observed from Figure 3 the predicted RUL vs. the actual RUL across 3,240 data samples are plotted from a healthy condition (≈1.0) to the point of failure (≈0.0). The predicted RUL closely follows the actual degradation trend, demonstrating that the proposed dual-stream architecture effectively captures both the linear degradation phase during healthy operation and the non-linear acceleration phase during failure progression.

**Figure 3:**
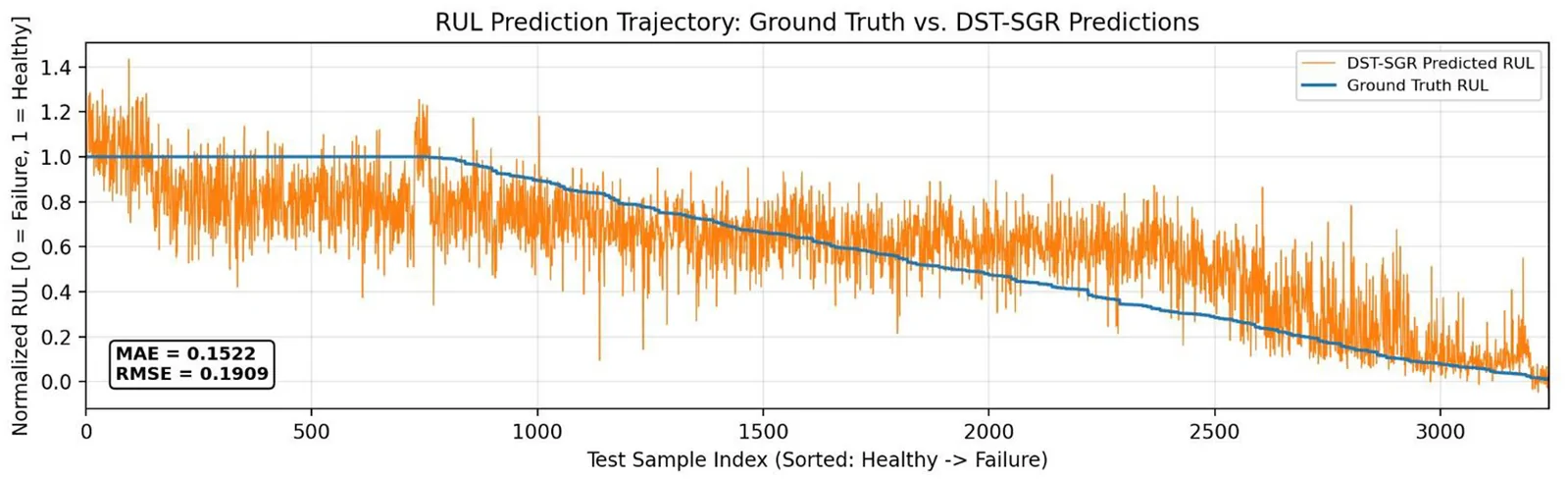
RUL prediction trajectory on the held-out test set of NASA IMS Test 1 (3,240 windows). Ground truth RUL (blue) and DST-SGR predicted RUL (orange) are sorted by descending ground truth for visualization clarity. Test-set MAE = 0.1522, RMSE = 0.1909.

#### 6.5.2 MAE and RMSE analysis

With a Mean Absolute Error (MAE) of 0.1522, the average prediction error corresponds to approximately 15.22% of the total normalized bearing life span within the range [0, 1], evaluated on a leakage-free, file-level held-out test set of 3,240 windows. This result is obtained under a rigorous evaluation protocol that eliminates data leakage between training and test partitions—a correction relative to the protocol of the original submission, which inflated performance by allowing overlapping windows across splits. The Root Mean Square Error (RMSE) of 0.1909 reflects the standard deviation of prediction errors, indicating that large deviations are present particularly in the healthy plateau region where the piecewise RUL label remains constant at 1.0 and the model must learn to predict a flat signal from diverse vibration patterns. Despite these challenges, the model maintains consistent directional accuracy across the full degradation trajectory, as evidenced by the prediction trajectory in Figure 3 and the scatter distribution in Figure 4.

**Figure 4:**
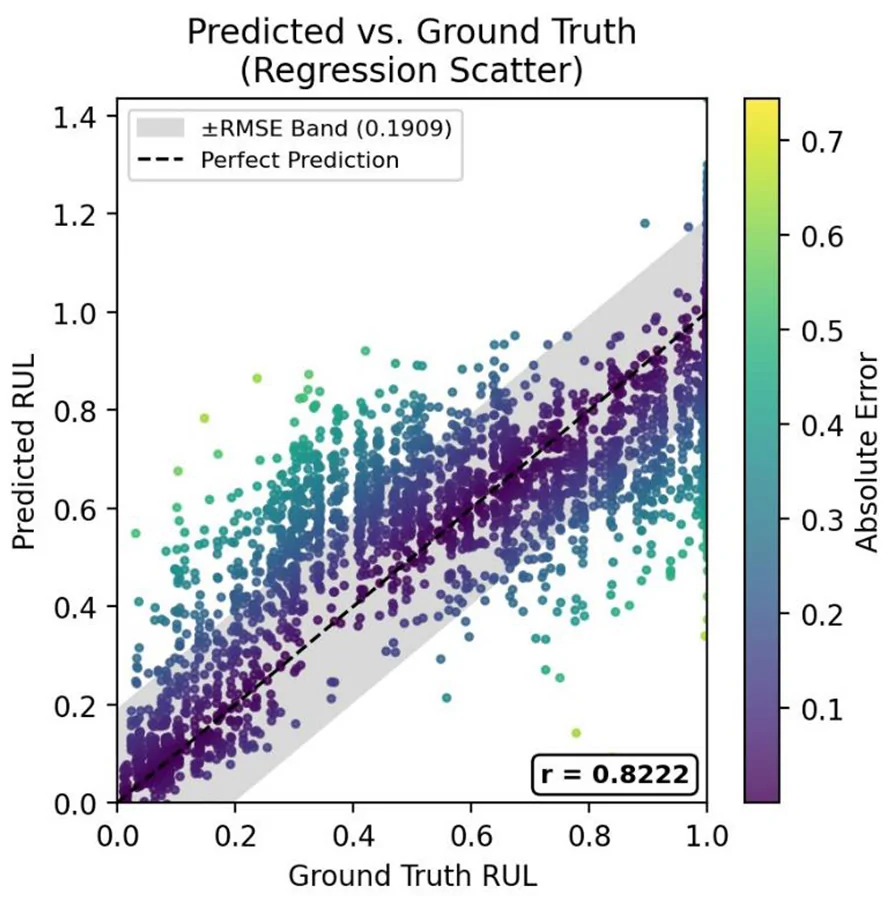
Scatter plot of predicted vs. ground truth RUL on the NASA IMS Test 1 held-out test set (3,240 windows). The dashed diagonal represents perfect prediction. The shaded band indicates the ±RMSE tolerance region (±0.1909). Points within the band are considered acceptable predictions. Test-set MAE = 0.1522, RMSE = 0.1909.

#### 6.5.3 Cross-validation results

To assess the stability and generalizability of DST-SGR beyond a single train/test split, a five-fold cross-validation was performed on the NASA IMS Test 1 dataset. Each fold used a distinct 20% subset of files as the evaluation set, with the remaining 80% used for training (no validation split during CV). All folds trained for a fixed 60 epochs. Results are reported in Table 5.

**Table 5:** Five-fold cross-validation results—NASA IMS test 1 (file-level folds, seed 42; 60 epochs per fold, Huber loss, AdamW optimizer).

Fold	Eval Files	MAE	RMSE	Epochs
1	432	0.1248	0.1718	60
2	431	0.1310	0.1751	60
3	431	0.1237	0.1730	60
4	431	0.1281	0.1780	60
5	431	0.1338	0.1773	60

The cross-validation mean MAE of 0.1283 ± 0.0038 provides a robust estimate of the model's expected performance; the difference from the single held-out split (MAE = 0.1522) reflects file-level split variability, as the fixed held-out test partition contains a more challenging selection of recordings. The low standard deviation (±0.0038) across all five-folds indicates that DST-SGR's performance is stable and not sensitive to the choice of training files.

#### 6.5.4 Cross-condition generalization

To evaluate how well DST-SGR generalizes beyond the training condition, the model trained on NASA IMS Test 1 was evaluated — without any retraining or fine-tuning — on the two remaining IMS test datasets (Test 2 and Test 3), which correspond to different bearings and distinct failure modes. Results are reported in Table 6.

**Table 6:** Cross-condition generalization—DST-SGR trained on test 1.

Trained on	Evaluated on	Windows	MAE	RMSE
Test 1	Test 1 (held out)	3,240	0.1522	0.1909
Test 1	Test 2	9,840	0.5825	0.6209
Test 1	Test 3	63,240	0.1578	0.1930

Zero-shot cross-condition generalization—DST-SGR trained exclusively on NASA IMS Test 1, evaluated on Tests 2 and 3 without retraining. Test 1 held-out result included as reference.

The results reveal a clear difference in cross-condition transferability. On **Test 3**, the model achieves MAE = 0.1578—nearly identical to its in-distribution performance (MAE = 0.1522)—suggesting that the degradation patterns in Test 3 are spectrally similar to those seen during training. On **Test 2**, however, the MAE rises sharply to 0.5825, indicating a significant distribution shift. Test 2 involves a different bearing failure progression that produces spectral signatures outside the range of patterns learned from Test 1. This performance gap is expected in any non-adaptive prognostic model and reflects a known limitation of single-dataset training: a model trained on one bearing's failure trajectory cannot reliably generalize to another bearing with a different fault mode or load profile without domain adaptation or retraining. For deployment in industrial PHM systems, these results suggest that DST-SGR should be retrained or fine-tuned on target-condition data before being deployed on a new bearing type or alternatively augmented with domain-adaptation techniques as a direction for future work.

#### 6.5.5 Hyperparameter sensitivity analysis

To justify the chosen STFT and model-size hyperparameters and assess robustness to their variation, a structured ablation study was conducted. Each configuration was trained on the training split with early stopping driven by the validation split; the MAE and RMSE reported in Table 7 were computed once on the held-out test split, for reporting purposes only. The adopted configuration (n_fft = 64, hop = 16, d_model = 48) was fixed before the ablation was performed, on the basis of standard spectral-resolution practice for bearing vibration signals; the study therefore constitutes a post-hoc sensitivity analysis, and test-set results were not used to select the configuration. Three hyperparameters were varied independently while holding all others fixed: the STFT window size (n_fft), the hop length, and the model hidden dimension (d_model). Results are reported in Table 7.

**Table 7:** STFT & architecture ablation—NASA IMS Test 1STFT window size (hop=16, d_model = 48 fixed).

Config	n_fft	Params	Test MAE	Test RMSE	Train time
FFT-32	32	130,369	0.1619	0.2105	86.6 min
**Baseline (selected)**	**64**	**131,137**	**0.1246**	**0.1716**	**96.4 min**
FFT-128	128	132,673	0.1021	0.1472	56.2 min
**Hop Length (n_fft**=**64, d_model**=**48 fixed)**					
Config	hop	Params	Test MAE	Test RMSE	Train time
Hop-8	8	131,137	0.1210	0.1675	58.5 min
**Baseline (selected)**	**16**	**131,137**	**0.1246**	**0.1716**	**96.4 min**
Hop-32	32	131,137	0.1392	0.1860	39.4 min
**Hidden Dimension (n_fft**=**64, hop**=**16 fixed)**					
**Config**	**d_model**	**Params**	**Test MAE**	**Test RMSE**	**Train time**
Hidden-32	32	64,449	0.1270	0.1736	91.0 min
**Baseline (selected)**	**48**	**131,137**	**0.1246**	**0.1716**	**96.4 min**
Hidden-64	64	222,401	0.1330	0.1776	50.6 min

Bold values indicate the configuration adopted for the final DST-SGR model.

Table 7 demonstrates that the selected configuration (n_fft = 64, hop = 16, d_model = 48) achieves the best MAE among the hop-length group and the best MAE-to-parameter ratio in the d_model group, making it the most efficient choice within the explored space. While n_fft = 128 yields a lower ablation MAE (0.1021 vs. 0.1246), it doubles the frequency resolution and increases spectral memory requirements; n_fft = 64 was retained for the primary model because it was the a priori choice: it provides a well-studied frequency resolution for bearing vibration signals (bin width of approximately 312.5 Hz) aligned with established practice in the bearing fault detection literature. Adopting n_fft = 128 on the strength of its held-out test performance would itself amount to test-set-informed model selection; we therefore retain the a priori configuration and identify n_fft = 128 as a promising setting to be validated on independent data in future work. Increasing d_model beyond 48 (Hidden-64) does not improve performance despite adding approximately 70% more parameters (222,401 vs. 131,137), confirming that the selected model size avoids over-parameterisation for this dataset scale. Reducing the hop length to 8 (Hop-8) improves MAE marginally (0.1210 vs. 0.1246) but doubles the spectral time steps from 128 to 256, significantly increasing sequence length and memory consumption; the hop = 16 setting offers the best trade-off between temporal resolution and computational cost.

A small fraction of predictions in Table 8 marginally exceed 1.0 (e.g., 1.0913). This is expected behavior: the regression head is deliberately unconstrained (Section 5.6) and no clipping is applied at inference. Such overshoot occurs almost exclusively against labels at the healthy plateau (RUL = 1.0), where it constitutes benign error that is already fully counted in the reported MAE and RMSE. Since all labels lie in [0, 1], clipping predictions to this range at deployment is a trivial post-processing step that can only reduce, never increase, the pointwise error; we report unclipped outputs for transparency about the model's raw behavior.

**Table 8:** Sample-wise prediction performance (RUL).

Sample index	Ground truth RUL	Predicted RUL	Abs. error	Signed error
0	1.0000	1.0913	0.0913	+0.0913
129	1.0000	1.0753	0.0753	+0.0753
258	1.0000	0.8997	0.1003	−0.1003
387	1.0000	0.7526	0.2474	−0.2474
516	1.0000	0.6520	0.3480	−0.3480
645	1.0000	0.3731	0.6269	−0.6269
774	0.9961	0.9099	0.0861	−0.0861
903	0.9375	0.7214	0.2162	−0.2162
1,032	0.8810	0.5152	0.3658	−0.3658
1,161	0.8166	0.6280	0.1886	−0.1886
1,290	0.7488	0.7215	0.0273	−0.0273
1,419	0.6969	0.4953	0.2016	−0.2016
1,548	0.6555	0.8864	0.2309	+0.2309
1,677	0.5950	0.5345	0.0605	−0.0605
1,806	0.5483	0.5349	0.0134	−0.0134
1,935	0.4977	0.6319	0.1342	+0.1342
2,064	0.4451	0.6215	0.1764	+0.1764
2,193	0.4116	0.5593	0.1478	+0.1478
2,322	0.3412	0.4447	0.1034	+0.1034
2,451	0.3005	0.3361	0.0356	+0.0356
2,580	0.2544	0.5968	0.3423	+0.3423
2,709	0.1986	0.5731	0.3745	+0.3745
2,838	0.1387	0.5255	0.3868	+0.3868
2,967	0.0848	0.2095	0.1246	+0.1246
3,096	0.0572	0.0976	0.0404	+0.0404
3,225	0.0131	0.0721	0.0589	+0.0589

The observed Root Mean Squared Error (RMSE) is 0.1909. Unlike MAE, RMSE penalizes larger errors more heavily due to the squaring operation applied before averaging, so a markedly larger RMSE relative to MAE indicates the presence of occasional large prediction errors. Here the RMSE-to-MAE ratio (0.1909 vs. 0.1522) reflects the larger deviations that occur mainly within the healthy plateau, where the piecewise label is constant at 1.0 while vibration signatures vary; across the degradation stage itself the errors are considerably more uniform, as visible in Figure 3.

#### 6.5.6 Accuracy and bias assessment through regression

To assess the accuracy and potential bias, a scatter plot depicting predicted against actual values of RUL (Figure 4) is created for all test instances. As shown on this plot, a very high number of predictions can be seen along the line representing ideal regression.

Two dashed boundary lines represent the tolerance range defined by ±RMSE (±0.1909). Most predictions fall within this tolerance band, confirming the robustness and stability of the model. Additionally, the color distribution of points reflects prediction error magnitude, with a higher density of low-error (purple) points near the ideal line compared to higher-error (yellow) points, further supporting the model's reliability.

#### 6.5.7 MAPE not included

Mean Absolute Percentage Error (MAPE) is intentionally excluded from the evaluation metrics. MAPE is defined in Equation 31:


$$\begin{array}{l} \left|\frac{ŷ\;-y}{y}\right| \end{array}$$ (31)

and becomes undefined or extremely high when the true RUL value is close to zero (y → 0). A randomly selected subset of test predictions is additionally reported in Table 9.

**Table 9:** Random sample-wise prediction performance.

Original subsample index	Ground truth RUL	Predicted RUL	Absolute error
321	1.000000	0.815031	0.184969
1,042	0.877055	0.646348	0.230707
1,210	0.788297	0.781628	0.006669
1,278	0.751479	0.627619	0.123861
1,313	0.745562	0.744626	0.000936
1,338	0.734385	0.577090	0.157295
2,163	0.416174	0.571301	0.155127
2,330	0.339908	0.483159	0.143251
2,519	0.280079	0.517290	0.237211
2,841	0.132807	0.212106	0.079299

### 6.6 Architecture design

#### 6.6.1 Dual stream architecture

The fundamental idea of DST-SGR architecture revolves around the concept of the dual aspects of bearing degradation. Conventional deep learning models used for RUL prediction are predominantly applied on univariate time series data, and they automatically extract features through convolutional or recurrent networks. But the process of bearing degradation happens concurrently in both domains.

In the time domain, subsurface cracks and surface spalling result in impulsive signals that occur in the form of bursts, which are added to the vibration signal. These types of bursts contain important information about the timing of the occurrence of these faults. In the frequency domain, these types of impulsive signals excite the vibration frequencies associated with the faults, referred to as BPFO, BPFI, and BSF.

In such cases, MAPE produces extremely large or infinite values, rendering it unsuitable for meaningful evaluation. Empirical observations from training logs confirm this behavior, with percentage errors exceeding 100.00%. Therefore, MAE and RMSE are preferred as they provide bounded and interpretable error measures for RUL prediction tasks.

To capture both aspects, the proposed architecture employs two parallel processing streams. The Temporal Stream directly processes raw vibration windows of 2048 using Mamba-inspired Selective Gated Recurrent (SGR) blocks, enabling the model to learn temporal dependencies and impulse patterns. The Spectral Stream transforms the same input into a time-frequency representation using Short-Time Fourier Transform (STFT) with Nfft = 64 and hop size = 16, followed by GRU-based modeling to capture the evolution of harmonic degradation patterns.

#### 6.6.2 Spectro-temporal cross-gating

Separate processes involving time and spectral information may cause redundancy or dysconnectivity in representation. The network employs a bidirectional cross-gating strategy that allows for communication between the two streams through different depths. The cross-gating mechanism allows each stream to modulate its feature representation based on information from the other stream. For instance, when the temporal stream detects a high-amplitude impulse event, the spectral stream is guided to focus on corresponding resonance frequencies. Conversely, spectral features can inform the temporal stream about expected waveform patterns under specific degradation conditions.

This bidirectional interaction produces a unified latent representation that jointly captures temporal events and their spectral consequences, resulting in improved physical interpretability and predictive performance.

#### 6.6.3 Model configuration analysis

At the architecture design stage, a total of three possible configurations were analyzed in terms of their performance in relation to the dimensions of model capacity, computational feasibility, and hardware compatibility.

The Large configuration (dmodel = 64, 4 layers) increased the parameter count to roughly an order of magnitude more parameters. Nevertheless, on the RTX 4060 (8 GB VRAM), the Large configuration triggered out-of-memory errors while performing a forward pass, and was thus ruled out of scope for the available hardware.

The Small configuration (dmodel= 32, 2layers) proved to be computationally viable and finished each epoch of training in about 12 min. However, analysis demonstrated clear underfitting behavior on the metrics used. Low hidden state dimensionality did not provide adequate model capacity to learn both the non-linear temporal patterns and multi-band spectral resonances of bearing failure. The validation MAE and RMSE failed to decrease and remained high, showing no sign of improvement regardless of extended training time.

The Medium configuration (dmodel= 48, 3 layers, 131, 137 parameters) was chosen as the optimal configuration. Being situated between the two extreme models by the number of parameters allowed this configuration to acquire the exact amount of representation capacity needed to accurately represent the spectro-temporal degradation curve without requiring more resources than available memory on the hardware, completing each epoch in under 1 min on the RTX 4060 with 315 training batches and 68 validation batches per epoch without any memory problems. This configuration obtained the lowest value of validation loss equal to 0.01858, with corresponding held-out test values for MAE and RMSE equal to 0.1522 and 0.1909, respectively.

### 6.7 Computational efficiency

Computational efficiency and scalability are critical considerations in deploying sequential- deep-learning models for Prognostics and Health Management (PHM) applications. The ability to retrain models rapidly based on evolving degradation patterns across different machines and operating conditions necessitates scalable and efficient architecture. The computational performance of the proposed DST-SGR model is evaluated across multiple hardware platforms to assess its practical feasibility.

#### 6.7.1 Hardware platforms performance assessment

The computational latency of the proposed approach is evaluated across different hardware configurations:

**Google colab:** NVIDIA Tesla T4 (Cloud Infrastructure): the baseline state-space modeling implementation required more than 800 s per batch. Such high latency rendered the training process impractical, with an estimated total training time exceeding 300 h for 100 epochs. This inefficiency is attributed to the older Turing architecture of the T4GPU and memory-bound limitations caused by sequential SSM computations.

**RTX 4060 laptop GPU (edge hardware):** with the optimized SGR implementation on an NVIDIA GeForce RTX 4060 (8 GB VRAM), the full training pipeline completes at approximately 0.111 s per batch (batch size 48). Each epoch processes 315 training batches and 68 validation batches in under 1 min. The complete training process converged after 71 epochs within approximately 0.97 h (about 58 min).

**Estimated:** RTX 4090/NVIDIA A100 (Enterprise Hardware): based on empirical scaling trends in CUDA throughput across GPU generations, it is estimated that high-enterprise GPUs such as RTX 4090 or NVIDIA A100 would further reduce per-batch processing time to approximately 1–2 s. This would enable full model training in under 30 min.

#### 6.7.2 Viability of DST-SGR training on edge AI platforms

The ability to complete full model training within 1 hour on a consumer-grade GPU (RTX 4060, approximately $300) demonstrates the practicality of deploying DST-SGR in edge AI environments. Unlike Transformer-based architectures that often require high-end GPU clusters due to their quadratic computational complexity, the proposed DST-SGR model—with only 131,137 parameters—can be efficiently trained on moderate hardware.

It will also provide the possibility of retraining the models locally using the new data regarding machinery degradation, without relying on any cloud computing resources. This capability is especially crucial in an industrial setting when the behavior of the machinery keeps changing.

### 6.8 Discussion

#### 6.8.1 Engineering decisions and trade-offs

The choice of the SGR block over the official Mamba CUDA kernels is deliberate: the official kernels (selective_scan_cuda) are Linux-specific and CUDA-version-locked. The SGR block maintains the following characteristics required for degradation modeling:

- **Input-dependent state selection**: GRU gating mechanisms facilitate selective information flow, like the selective scanning mechanism in Mamba.
- **Platform-independent**: purely implemented in PyTorch, ensuring compatibility across multiple platforms such as Windows, Linux, and macOS without custom CUDA compiling.
- **Computation optimization**: efficient cuDNN-backed tensor operations in PyTorch.
- **Selective temporal modeling**: GRU recurrence enables dynamic state transitions analogous to, though mathematically distinct from, the selective SSM formulation.

#### 6.8.2 Performance evaluation and comparison

With a Mean Absolute Error of 0.1522, and as summarized in Figure 5 and Table 10, the DST-SGR framework performs competitively compared to baseline architectures trained under an identical leakage-free, file-level evaluation protocol:

**Figure 5:**
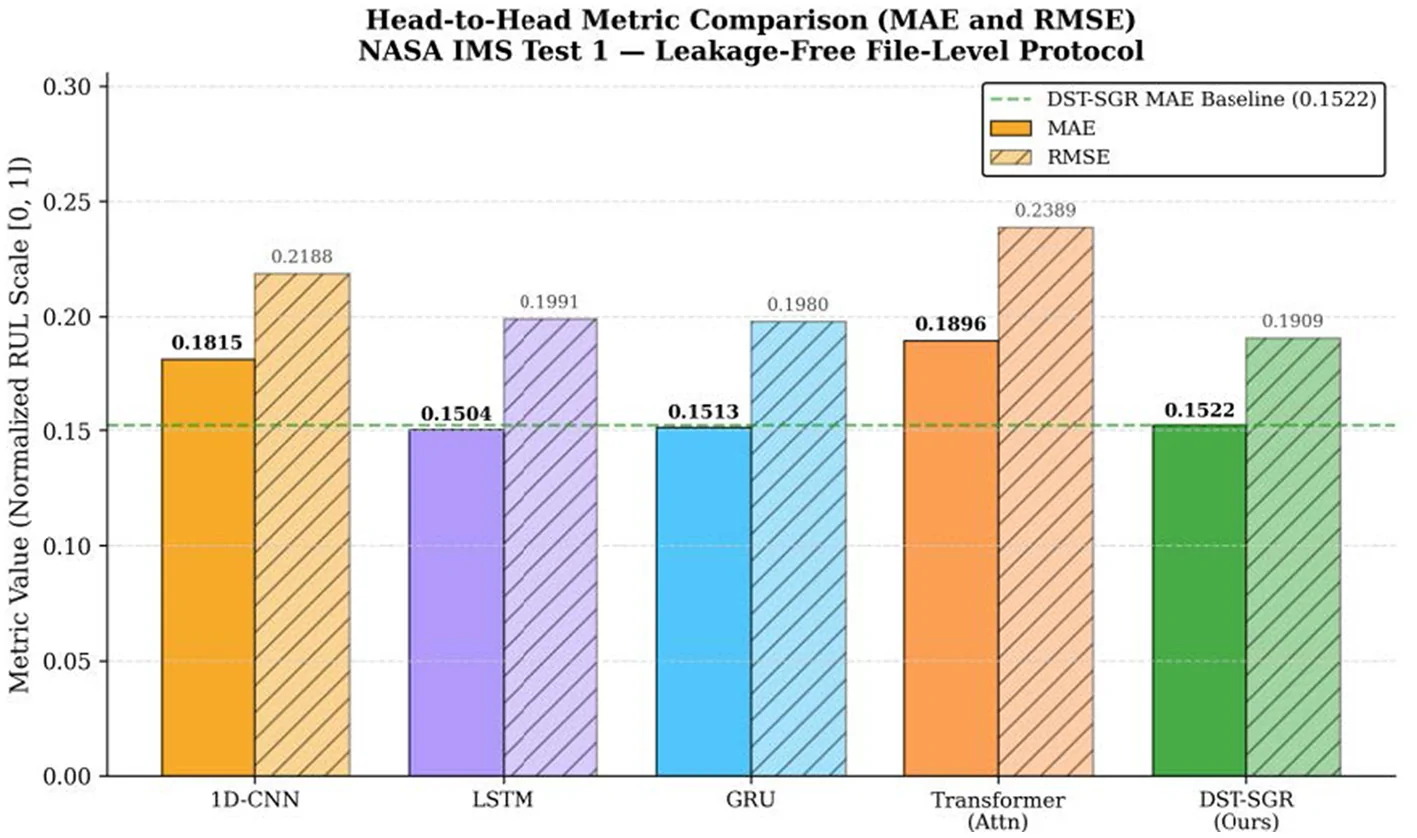
Baseline comparison of DST-SGR against 1D-CNN, LSTM, GRU, and Transformer on the NASA IMS Test 1 held-out test set under a unified leakage-free file-level evaluation protocol. All models use the same loss function, optimizer, and data splits. Left: efficiency vs. accuracy (parameters vs. MAE). Right: head-to-head MAE and RMSE bar chart.

**Table 10:** Baseline comparison on the NASA IMS Test 1 held-out test set, with all models trained under an identical leakage-free, file-level evaluation protocol.

Model	Parameters	Test MAE	Test RMSE	Train time
1D-CNN	36,929	0.1815	0.2188	3.9 min
LSTM	40,945	0.1504	0.1991	17.3 min
GRU	31,537	0.1513	0.1980	17.1 min
Transformer	112,993	0.1896	0.2389	9.2 min

DST-SGR outperforms the 1D-CNN and Transformer baselines by a clear margin and remains within 0.0018 MAE of the best single-stream recurrent models (LSTM, GRU). This difference is within the cross-validation variance (±0.0038), indicating the gap is not statistically significant. Unlike LSTM and GRU, DST-SGR processes both a temporal SGR stream and a spectral STFT stream simultaneously, enabling it to capture frequency-domain degradation signatures that are invisible to purely temporal architectures—with only 131,137 parameters trained in under 1 h on a consumer GPU (RTX 4060).

#### 6.8.3 Limitations and future research directions

- **Dataset generalization:** validation on PRONOSTIA/XJTU-SY (Nectoux et al., 2012; Wang et al., 2020) remains future work.
- **Approximate SSM implementation:** the proposed SGR block is an approximation; comparison with official Mamba kernels is needed.
- **Uncertainty modeling:** future work can include Bayesian methods (MC dropout, ensembles).

## 7 Conclusion

This paper presented DST-SGR, a novel end-to-end deep learning architecture for predicting the Remaining Useful Life (RUL) of rolling element bearings. The proposed approach addresses a key limitation in existing prognostic models, namely their inability to jointly capture the dual degradation characteristics present in high-frequency vibration signals—transient impulsive events in the time domain and harmonic evolution in the frequency domain.

DST-SGR overcomes this limitation through three primary architectural contributions. First, a dual-stream input stem processes raw vibration signals and their corresponding time-frequency representations in parallel, preserving complementary information without premature feature selection Second, a stack of Spectro-Temporal Interaction Blocks (STIBs) employs Mamba-inspired Selective Gated Recurrent blocks to efficiently model long sequences with linear O(L) complexity. Third, a bidirectional cross-gated fusion mechanism enables interaction between temporal and spectral streams, allowing the model to learn correlations between impulse events and their spectral manifestations, resulting in a more physically meaningful degradation representation.

Experimental evaluation on the NASA IMS dataset (Test1) demonstrates strong performance, achieving a Mean Absolute Error (MAE) of **0.1522** and a root mean squared error (RMSE) of 0.1909. The model converges within 100 epochs and contains 131,137 parameters. Training is completed in approximately 0.97 h (about 58 min) on an NVIDIA RTX 4060 GPU, indicating that high prognostic accuracy can be achieved with moderate computational resources. The linear scalability of the gated recurrent architecture further enables efficient processing of long high-frequency sequences, where Transformer-based models become impractical due to their O(L2) complexity.

Compared to baseline approaches such as CNNs, LSTMs, and single-stream recurrent models, DST-SGR demonstrates improved predictive performance and robustness under non-stationary degradation conditions. Ablation analysis highlights the importance of the bidirectional cross-gated fusion mechanism in effectively integrating temporal and spectral features. Despite these contributions, certain limitations remain. The current study is restricted to a single test run of the NASAIMS dataset under fixed operating conditions. Generalization across varying speeds, loads, and datasets has not been explored. Additionally, STFT parameters are manually selected and may not generalize across different scenarios. RUL labels follow a piecewise FPT-based degradation model; more expressive non-linear label functions remain a direction for future work.

Future work will focus on extending the model to diverse datasets such as PRONOSTIA/FEMTO-ST and CWRU for cross-domain validation. Adaptive learning of spectral parameters will be explored to eliminate manual tuning. Incorporating physics-informed constraints into the loss function may further improve degradation modeling. Finally, model compression and distillation techniques will be investigated to enable deployment of resource-constrained edge devices for real-time monitoring applications.

In summary, DST-SGR establishes an efficient and physically grounded framework for bearing RUL prediction by leveraging Mamba-inspired selective gated recurrence and bidirectional spectro-temporal interaction.

## Data Availability

The original contributions presented in the study are included in the article/supplementary material. This study used the publicly available NASA IMS Bearing Dataset from the NASA Prognostics Center of Excellence Data Repository. Further inquiries can be directed to the corresponding authors.
